# Engineering mannose-functionalized nanostructured lipid carriers by sequential design using hybrid artificial intelligence tools

**DOI:** 10.1007/s13346-024-01603-z

**Published:** 2024-05-09

**Authors:** Rebeca Martinez-Borrajo, Patricia Diaz-Rodriguez, Mariana Landin

**Affiliations:** Departamento de Farmacología, Farmacia y Tecnología Farmacéutica, Grupo I+D Farma (GI-1645), Facultad de Farmacia, Instituto de Investigación Sanitaria de Santiago de Compostela (IDIS), Instituto de Materiais da Universidade de Santiago de Compostela (iMATUS), Universidade de Santiago de Compostela, 15782 Santiago de Compostela, Spain

**Keywords:** Nanostructured lipid carriers, Carbohydrate surface functionalization, Artificial intelligence, Quality by design, Mannosylation optimization, Artificial neural networks, Genetic algorithms, Neurofuzzy logic

## Abstract

Nanostructured lipid carriers (NLCs) hold significant promise as drug delivery systems (DDS) owing to their small size and efficient drug-loading capabilities. Surface functionalization of NLCs can facilitate interaction with specific cell receptors, enabling targeted cell delivery. Mannosylation has emerged as a valuable tool for increasing the ability of nanoparticles to be recognized and internalized by macrophages. Nevertheless, the design and development of functionalized NLC is a complex task that entails the optimization of numerous variables and steps, making the process challenging and time-consuming. Moreover, no previous studies have been focused on evaluating the functionalization efficiency. In this work, hybrid Artificial Intelligence technologies are used to help in the design of mannosylated drug loaded NLCs. Artificial neural networks combined with fuzzy logic or genetic algorithms were employed to understand the particle formation processes and optimize the combinations of variables for the different steps in the functionalization process. Mannose was chemically modified to allow, for the first time, functionalization efficiency quantification and optimization. The proposed sequential methodology has enabled the design of a robust procedure for obtaining stable mannosylated NLCs with a uniform particle size distribution, small particle size (< 100 nm), and a substantial positive zeta potential (> 20mV). The incorporation of mannose on the surfaces of these DDS following the established protocols achieved > 85% of functionalization efficiency. This high effectiveness should enhance NLC recognition and internalization by macrophages, thereby facilitating the treatment of chronic inflammatory diseases.

**Graphical Abstract:**

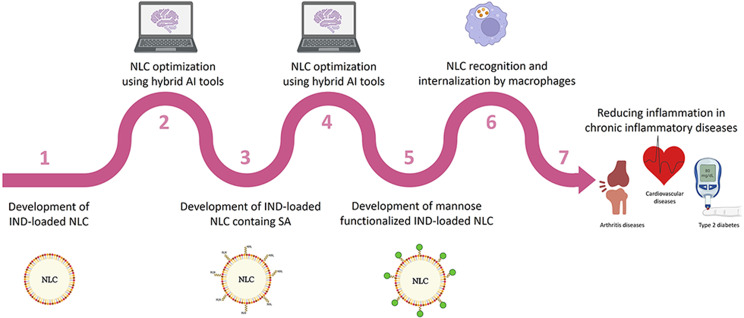

**Supplementary Information:**

The online version contains supplementary material available at 10.1007/s13346-024-01603-z.

## Introduction

Nanoparticles (NPs) have several advantages as drug delivery systems (DDS) due to their particle size, allowing them to cross human biological barriers. This unique characteristic converts these systems in versatile tools to prevent or treat different pathologies [8, 31]. NPs can be developed employing a variety of techniques including nanoprecipitation and emulsion, and using different raw materials such as inorganic (metals and ceramics), or organic (lipids and polymers) ingredients [8, 31].

Nanostructured lipid carriers (NLCs) are organic nanoparticles composed of liquid and solid lipids and surfactants [29]. Some of their main advantages are the highly efficient drug loading, controlled drug release, and enhanced drug stability. These can be primarily attributed to their imperfect matrix, which significantly increases the available space for drug incorporation [8, 29, 31].

Moreover, NLC surface can be coated or functionalized with certain components (carbohydrates such as glucose, mannose, galactose… antibodies, vitamins…), that can lead to targeted systems. Functionalized NLC will show interaction with specific cell receptors, improving cell selectivity and uptake [3, 15, 19].

Human mannose receptor (CD206 or MRC1) is a glycoprotein expressed on the membrane of almost all macrophages, including tissue specific macrophages. This membrane receptor exhibit capacity to recognize pathogens comprising N-acetylglucosamine, mannose and fucose residues on their surface, such as bacteria or fungi [6, 7]. Moreover, CD206 plays a role in homeostasis, infections, and inflammatory responses [7]. Given the essential role of macrophages in inflammation control, they emerge as crucial players in chronic inflammatory diseases as type 2 diabetes, cardiovascular or autoimmune diseases, and arthritis among others [16, 20, 27]. The development of mannose-functionalized DDS may be a good strategy to ensure their recognition and internalization by macrophages. This innovative approach holds potential for designing DDS tailored to effectively manage inflammation-related pathologies.

However, the development of NLC is already a complex task where many variables are involved (ingredients and operation conditions). It is even more challenging when additional steps are required for functionalization. The complexity of establishing the nanocarriers design space can be addressed using artificial intelligence (AI) tools. This approach has been proven to be useful to predict and even optimize formulation processes from a set of experimental data, pointing out the relationships between inputs (composition and operation variables) and outputs (final nanoparticles characteristics) [5, 14, 24].

Nevertheless, the development of carbohydrate functionalized NLC requires the addition of further components (functionalization linkers). The incorporation of these extra ingredients entails the modification of the NLC composition and properties, demanding additional studies to ensure the formation and stability of the modified NLC. Generally, these variations towards functionalization are not considered in the optimization process, instead, optimized systems are obtained and subsequently, functionalized following a trial-and-error strategy [22]. This approach, focused primarily on the functionalization effect on NPs physicochemical properties (size and surface charge), does not ensure the functionalized NPs show the best drug loading and functionalization efficiency. Therefore, the study of the additional components effect on drug loading and functionalization based on tracking the carbohydrates incorporation can be an adequate tool to obtain optimal functionalized NLC. However, there is no commercially available labelled mannose that can be used to this end. Consequently, based on the Maillard reaction, we have attached a fluorescent dye to the mannose backbone and characterized the modified carbohydrate. The use of this labelled molecule will allow for the improvement of the functionalized NLC ensuring the adequate bond formation between the targeting ligand, mannose, and the amino groups of the linker present in the NLC surface. These systems will not only show adequate physicochemical properties but also high drug incorporation and functionalization efficiency.

Thus, the main objective of this work is to evaluate the utility of hybrid AI tools (artificial neural networks combined with fuzzy logic or genetic algorithms) and fluorescently labelled mannose to assist in the development of stable carbohydrate-functionalized drug-loaded NLC. The anti-inflammatory drug indomethacin (IND) was selected as the cargo for the NLC. The proposed sequential strategy aims to build a database incorporating the results obtained in each step of the process to achieve a model that allows establishing the final design space for functionalized and stable NLC systems. In this way, four steps will be followed: (1) Design of indomethacin (IND)-loaded NLC with adequate physicochemical properties and drug loading by selecting the suitable composition and production procedure; (2) Design of IND-loaded NLC containing the functionalization linker ensuring suitable drug loading, size, and polydispersity index; (3) Design of IND-loaded NLC containing the functionalization linker with a high drug payload and stable for at least 3 months; (4) Design of carbohydrate functionalized IND-loaded NLC showing the desired properties of step 3 together with high functionalization efficiency. The functionalization efficiency will be studied by synthesizing anthranilic acid labelled mannose. To the best of our knowledge, the use of fluorescently labelled carbohydrates has never been used for developing functionalized NPs.

## Materials and methods

### Materials

Indomethacin (IND) was purchased from Acros Organics (Geel, Belgium). Transcutol^®^ P (highly purified diethylene glycol monoethyl ether), Capryol^®^ 90 (propylene glycol monocaprylate), Labrafac™ lipophile WL 1349 (triglycerides medium-chain), Labrafac™ PG (propylene glycol dicaprylate), Labrafil^®^ M 2125 CS (linoleoyl macrogol-6 glycerides), Labrafil^®^ M 1944 CS (oleoyl macrogol-6 glycerides), and Labrasol^®^ ALF (caprylocaproyl macrogol-8 glycerides) were used as liquid lipids (LL). Compritol^®^ 888 ATO (glyceryl behenate), and Precirol^®^ ATO 5 (glyceryl distearate) were selected as solid lipids (SL). Both LL and SL were kindly provided by Gattefossé (Saint-Priest, France). Epikuron^®^ 145 V (lecithin) (deoiled phosphatidyl choline-enriched lecithin) was a kind gift from Cargill (Wayzata, MN, USA). Polysorbate 80 (Tween^®^ 80), dialysis membrane (Spectrum. Labs Spectra/Por, MWCO 3.5 kDa), octadecylamine (stearylamine) (SA) and the D- (+)-Mannose were acquired from Sigma Aldrich (St Louis, MO, USA). Sodium cyanoborohydride 95% and Anthranilic acid 98% were acquired from Acros Organics (Geel, Belgium). Acetate buffer at pH 4 was prepared using acetic acid 0.2 M from Sigma Aldrich (St Louis, MO, USA) and sodium acetate 0.2 M from Scharlab (Barcelona, Spain). Ultrapure water (Milli-Q^®^ plus, Millipore Ibérica, Madrid, Spain) was used throughout all the experiments and the remaining solvents and reagents were analytical or HPLC grade.

### Selection of liquid and solid lipids

To select the most appropriate liquid lipid (LL) for the indomethacin encapsulation (IND), a solubility study was conducted following a procedure described previously by Gaspar et al. with slight modifications. To this end, 200 mg of IND were added to 1 mL of each LL and stirred for 48 h at 300 rpm. Then samples were centrifuged at 12,000 rpm and 20 °C for 30 min to remove drug crystals [10]. Subsequently, samples were suitably diluted in acetonitrile, and the IND concentration was quantified spectrophotometrically (Agilent Technologies, Spain) at 322 nm. Solubility studies were carried out in triplicate.

The most suitable solid lipid (SL) was selected by evaluating the solubility of IND in Compritol^®^ 888 ATO (melting range between 65 °C and 77 °C) and Precirol^®^ ATO 5 (melting range between 50 °C and 60 °C). To accomplish this, aliquots of each SL, weighing 200 mg were heated in a water bath at 80 °C until fully melted. Gradually increased amounts of IND were added to each lipid until a precipitate of non-solubilized drug became visible. The solubility of IND in the SL is expressed as the maximum amount that can be solubilized without the presence of precipitate [10].

Finally, the miscibility of the selected LL and both SL was examined blending them in various proportions (75:25, 50:50, 25:75). The mixtures were then heated in a water bath at 80 °C for 5 min. The presence of phase separation upon observation indicated lack of miscibility.

### NLC formulation and design

**Fig. 1:**
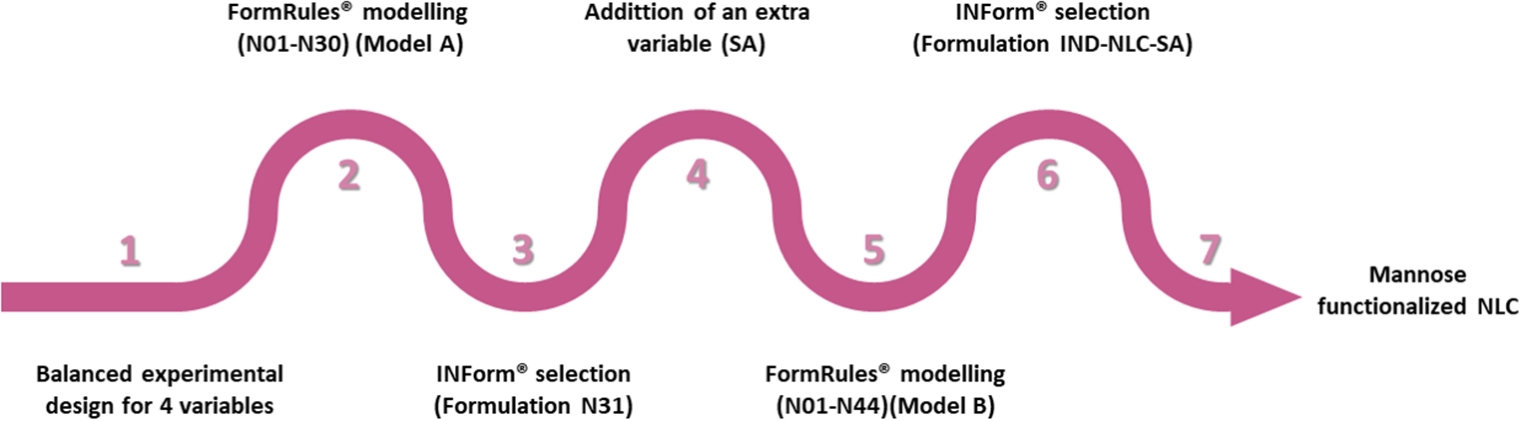
Stepwise optimization procedure to obtain optimized mannose-functionalized nanostructured lipid carriers (NLC) using hybrid artificial intelligence (AI) tools

NLC were prepared using hot shear homogenization similar to previously reported [25]. In brief, a lipid phase (300 mg) comprising the selected lipids with or without indomethacin (Transcutol^®^ P and Compritol^®^ 888 ATO) was prepared. Simultaneously, a separate aqueous phase (10 mL) containing lecithin and Tween^®^ 80 was also prepared. Both phases were obtained as specified in Table 1 and then heated in a water bath at 80 °C for 5 min. Subsequently, the aqueous phase was added to the lipid phase, and the mixture was homogenized for 10 min at 14,800 rpm using an Ultra-Turrax T25 (IKA Labortechnik, Staufen, Germany). The obtained dispersion was rapidly cooled in an ice bath with gentle agitation for 2 min. Each formulation was characterized before and after dialysis (MWCO 3.5 kDa) for the time specified in Table 1 to eliminate the non-incorporated components [24, 25]. A reduced experimental design for four variables (LL/SL ratio, Tween^®^ 80 concentration, amount of lecithin, and dialysis time) with a minimum pattern of 3 was established using DataForm^®^ v3.1 software (Intelligensys Ltd, UK). Both blank (unloaded) and IND loaded NLC formulations were prepared as indicated in Table 1 (Formulations N01 to N30). The amount of drug used for each loaded formulation was the maximum not leading to a drug precipitate. The obtained database (highlighted in grey) was modelled (Model A) and the obtained model was then validated (**Formulation N31**). In a second step, as described in Fig. 1, a new variable, stearylamine (SA), required for carbohydrate functionalization to endow the NPs with moieties able to covalently link these components, was incorporated [17, 30]. These new formulations were prepared adding SA to the lipid blend following the conditions shown in Table 1 (Formulations N32 to N44). The increased database was modelled again (Model B) (Formulations N01 to N44), the obtained model was re-validated (**IND-NLC-SA**), and the stability of the formulation was assessed after three months of storage. Finally, the stable formulation containing the functionalization linker was covalently functionalized with mannose though the Maillard reaction and characterized.

**Table 1:** Formulations prepared for Model **A** (grey) and Model **B** (white) along with the distinctive composition characteristics and purification conditions

Formulation	LL/SL Ratio (%)	Tween^®^ 80 (%)	Lecithin (mg)	Dialysis time (h)	IND amount (mg)	SA amount (mg)
N01	50.0	0.5	2.0	12.3	0.0	0.0
N02	30.0	2.0	1.5	0.5	0.0	0.0
N03	10.0	1.3	1.0	24.0	0.0	0.0
N04	10.0	1.3	2.0	12.3	0.0	0.0
N05	50.0	0.5	1.0	0.5	0.0	0.0
N06	30.0	2.0	1.5	24.0	0.0	0.0
N07	30.0	0.5	2.0	12.3	0.0	0.0
N08	10.0	2.0	1.5	24.0	0.0	0.0
N09	50.0	1.3	1.0	0.5	0.0	0.0
N10	30.0	2.0	1.0	24.0	0.0	0.0
N11	50.0	1.3	2.0	12.3	0.0	0.0
N12	10.0	0.5	1.5	0.5	0.0	0.0
N13	50.0	2.0	1.0	24.0	0.0	0.0
N14	10.0	0.5	2.0	0.5	0.0	0.0
N15	30.0	1.3	1.5	12.3	0.0	0.0
N16	50.0	0.5	2.0	12.3	6.1	0.0
N17	30.0	2.0	1.5	0.5	14.5	0.0
N18	10.0	1.3	1.0	24.0	4.8	0.0
N19	10.0	1.3	2.0	12.3	4.8	0.0
N20	50.0	0.5	1.0	0.5	5.9	0.0
N21	30.0	2.0	1.5	24.0	14.4	0.0
N22	30.0	0.5	2.0	12.3	4.4	0.0
N23	10.0	2.0	1.5	24.0	4.8	0.0
N24	50.0	1.3	1.0	0.5	12.3	0.0
N25	30.0	2.0	1.0	24.0	14.3	0.0
N26	50.0	1.3	2.0	12.3	6.3	0.0
N27	10.0	0.5	1.5	0.5	4.8	0.0
N28	50.0	2.0	1.0	24.0	18.2	0.0
N29	10.0	0.5	2.0	0.5	4.6	0.0
N30	30.0	1.3	1.5	12.3	10.8	0.0
N31	50.0	1.7	2.0	2.0	16.5	0.0
N32	10.0	0.5	2.0	0.5	4.6	4.3
N33	10.0	0.5	2.0	0.5	4.6	7.7
N34	50.0	0.5	1.0	0.5	5.9	4.4
N35	50.0	0.5	1.0	0.5	6.0	7.7
N36	50.0	1.7	2.0	2.0	16.3	1.4
N37	50.0	1.7	2.0	2.0	17.2	4.2
N38	50.0	1.7	2.0	2.0	16.4	6.3
N39	48.1	1.8	1.5	3.0	12.2	0.0
N40	48.1	1.8	1.5	3.0	11.7	7.7
N41	48.1	1.8	1.5	3.0	11.7	0.6
N42	48.1	1.8	1.5	3.0	11.5	1.7
N43	48.1	1.8	1.5	3.0	11.2	4.5
N44	48.1	1.8	1.5	3.0	11.7	6.9

### Mannose labelling and NLC functionalization

Mannose was labelled with anthranilic acid (2-aminobenzoic acid) (2AA) through a Maillard reaction similarly to already reported [11]. Briefly, 6 mg of anthranilic acid (2AA) was dissolved in 100 µL of dimethyl sulfoxide: acetic acid (7:3, v: v) containing 1 M of sodium cyanoborohydride (NaBH_3_CN). Then, mannose (25.2 mg) was dissolved in the previously prepared solution. The resulting mixture was vigorously vortexed at maximum speed (Fisherbrand, USA), protected from light, and incubated for 12 h at 37 °C. After this time, the labelled mannose was diluted in acetate buffer, purified employing a cyano-modified silica gel-SPE column (Chromabond^®^), and lyophilized.

Proton nuclear magnetic resonance spectroscopy analysis (^1^H-NMR) was conducted to confirm the labelling. For this purpose, fluorescently labelled mannose and unlabelled mannose were dissolved in deuterated water (D_2_O) obtained from Sigma Aldrich (St Louis, MO, USA) at 200 mM, while anthranilic acid was dissolved in deuterated dimethyl sulfoxide (DMSO-D6; Cambridge Isotope Laboratories, Inc. (Andover, MA, USA)) at the same concentration. The solutions were then placed in NMR tubes (600 µl) to be characterized using a Bruker NEO-750 equipment (Karlsruhe, Germany).

### NLC functionalization

Drug loaded NLC suspensions containing SA (**IND-NLC-SA**) were incubated with 50 mM fluorescently labelled D-(+)-mannose solution (MAN) in acetate buffer at pH 4. The mixtures were kept under magnetic stirring at 300 rpm (Fisherbrand, USA) protected from light at different timepoints. Subsequently, to eliminate unreacted components, formulations underwent a 12-hour dialysis using a cellulose membrane (MWCO: 3.5 kDa) under stirring in Milli-Q^®^ water.

Various molar ratios of SA: MAN (1:2, 1:3, and 1:5) and incubation times (24 h, 48 h, and 72 h) were tested to select the best functionalization strategy. To assess the effectiveness of the functionalization, the non-linked MAN was quantified. For this purpose, formulations underwent ultrafiltration using Amicon^®^ 100 kDa ultrafilters (Sigma Aldrich) at 10,000 g and 4 °C for 15 min. Subnatants were collected, and fluorescence was measured at 360 nm/425 nm using a plate reader (FLUOstar Omega, BMG Labtech, Germany). A calibration curve with MAN was also included during the fluorescence determination.

### NLC characterization

NLC were characterized in terms of particle size (size), polydispersity index (PDI), and surface charge (ZP) using a Zetasizer Pro (Malvern Instruments, Malvern, UK). Samples were diluted with Milli-Q^®^ water (1:10), placed in a specific cuvette (DTS 1070), and measured in triplicate using the parameters automatically selected by the software. All the measurements were performed at 25 °C ± 1 °C.

Additionally, the morphology of **IND-NLC-SA** nanoparticles functionalized with D-(+)-mannose at 1:2 SA: MAN ratio for 72 h (**IND-NLC-SA-MAN**) was evaluated through Transmission Electron Microscopy (TEM). Colloidal dispersions were placed onto cooper grids coated with a carbon membrane and stained with 2% (w/v) phosphotungstic acid solution for 2 min, following a previously described procedure [26]. The observation was conducted using a JEOL microscope (JEM 2010, Tokyo, Japan) equipped with a Gatan OriusTM camera (Gatan, Inc., Pleasanton, CA, USA).

Drug loading (DL) within the lipid network after purification (dialysis) of each formulation was calculated using Eq. 1, before and after storage. To determine actual drug content, samples were dissolved in acetonitrile and centrifuged at 12,000 rpm and 4 °C for 30 min. The supernatants were filtered through 0.45 μm and properly diluted in acetonitrile. IND quantification was performed spectrophotometrically at 322 nm.

$$DL \left(\%\right)=\left[\frac{\text actual \, drug \, content\left(mg\right)}{\text weight \, of \, nanoparticles \left(mg\right)}\right] x 100$$

(1)

### NLC modeling and optimization

The NLC characterization data of the first-step formulations (Table S1; N01-N30) was modelled using FormRules^®^v4.03 (Intelligensys Ltd., UK) (Model A). FormRules^®^ is a commercial software that combines two AI techniques, artificial neural networks (ANN) and fuzzy logic. This software uses the Adaptive Spline Modelling of Data (ASMOD) algorithm to generate models that relate inputs (ingredients and operation conditions) and outputs (formulation properties). Its ability to generate IF-THEN linguistic rules makes easier to understand the effects of the different ingredients and operating conditions on the process or products produced. This allows generating knowledge about the key variables to obtain IND-loaded NLC. FormRules^®^ enables the use of different fitting statistical criteria. Among them, Structural Risk Minimization (SRM) was selected as it provided the highest predictability along with the simplest and most intelligible rules. Five variables were introduced as inputs: LL/SL ratio, Tween^®^ 80 concentration, amount of lecithin, dialysis time, and the amount of IND. On the other hand, four parameters were included as outputs: Size, PDI, ZP, and DL.

The training parameters used by FormRules^®^ software were the following: ridge regression factor of 1.0 e^− 6^, number of set densities: 2, set densities: 2 and 3, maximum inputs per submodel: 2, maximum nodes per input: 15, adapt nodes: true, C_1_ values over 0.75.

The determination coefficient of the Training set (R^2^) (Eq. 2) was used to establish the predictability of the models, where *y*_*i*_ is the actual value in the data set, *y*_*i*_*´* is the value calculated by the model, and *y*_*i*_*”* is the mean of the dependent variable. The greater the value of the training set R^2^, the higher the predictability of the model.

$$ {R}^{2}= \left[1-\frac{{\sum _{i=1}^{n} ({y}_{i}-{y}_{i})}^{2}}{{\sum _{i=1 }^{n}({y}_{i}-{y}_{i})}^{2}}\right]x 100\%$$ (2)

The comparison between predicted and experimental values has been conducted using an ANOVA. The absence of differences between both sets indicates the accuracy of the model.

On the other hand, INForm^®^v5.01 software (Intelligensys Ltd, United Kingdom), integrates two AI technologies, ANN and Genetic Algorithms (GA), and was specifically designed for optimizing pharmaceutical formulations. INForm^®^ was used to obtain an optimized formulation using the initial dataset (**Formulation N31**). The training parameters used for the models were: 1 hidden layer, 2 nodes, transfer type (Asymmetric Sigmoid), output transfer type (Asymmetric Sigmoid), back propagation type (RPROP), target interactions (2000), target mean squared error (0.001), and random seed (10,000) and the optimization parameters were: number of populations = 1, number of interactions = 100, population size = 100, percentage of replacement = 50, mutation standard deviation = 0.1, and random seed = 1.

A second model (Model B) was developed using the data from formulations with and without the functionalization linker (Table S1; N01-N44). FormRules^®^ was employed to model the database under the same conditions as previously described, adding the amount of SA (mg) as an additional input.

Based on these models, a protocol (composition and appropriate purification conditions) for suitable and stable NLC including the functionalization linker was established. Specifically, the protocol aimed at obtaining NLC with small particle size (< 120 nm), maximum surface charge, and the highest drug loading (> 2%) not showing differences in their physicochemical properties after three months of storage. For this purpose, INForm^®^ was again employed. The training parameters used for the models were: 1 hidden layer, 3 nodes, transfer type (Asymmetric Sigmoid), output transfer type (linear), Back propagation type (RPROP), target interactions (1000), target mean squared error (0.0001), and random seed (10,000).

### Statistical analysis

The obtained data are expressed as mean ± SD and analyzed using GraphPad Prism 8 software. The groups were compared by performing one-way analysis of variance (ANOVA) followed by post hoc Tukey’s Multiple Comparison Test. The confident interval was 95%.

## Results and discussion

### LL and SL selection

**Fig. 2:**
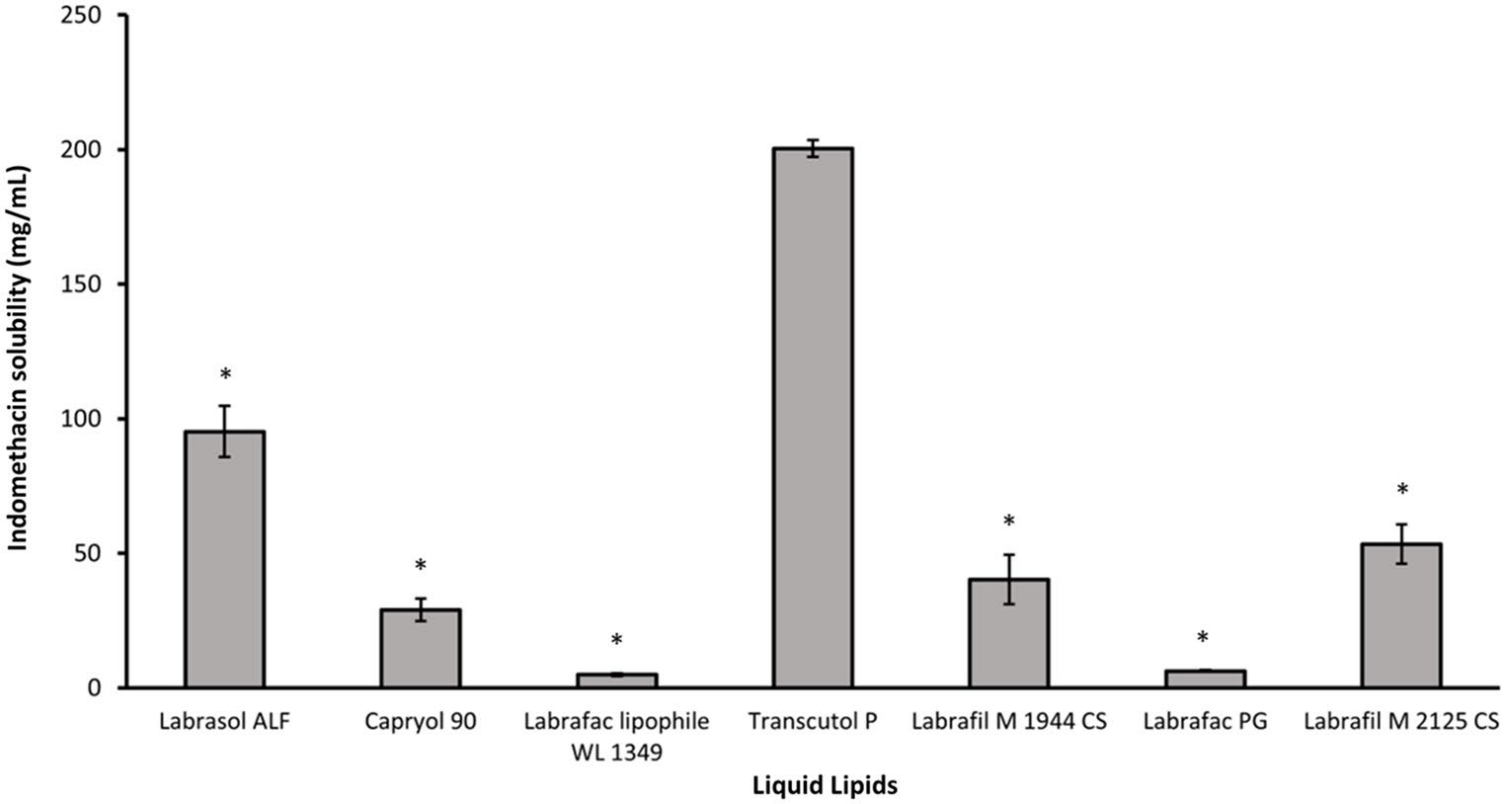
Solubility of indomethacin (IND) in the different liquid lipids (LL). Values are mean ± SD (*p < 0.0001 vs. Transcutol^®^*P*)

The IND solubility in different liquid lipids (LL) is presented in Fig. 2. Based on the obtained results, Transcutol^®^ P (HLB not applicable; logP = 0.5) was selected as the LL for the development of the NLC. The solubilization capacity for Transcutol^®^ P (197.22 ± 3.12 mg/mL) compared to all the other LL was significantly higher (*p* < 0.0001). On the other hand, Labrafac™ lipophile WL 1349 and Labrafac™ PG (HLB = 1) were the LL showing the lowest solubilization capacity. No significant differences in solubility were observed between Capryol^®^ 90 (HLB = 5), Labrafil^®^ M 2125 CS (HLB = 9), and Labrafil^®^ M 1944 CS (HLB = 9). IND presents also a high solubilization capacity (85.97 ± 9.46 mg/mL) in Labrasol^®^ ALF (HLB = 12). These differences in solubility can be mainly associated with interactions between the drug and the lipid. Despite drugs with high logP usually dissolve better in substances with low HLB value, the molecular structure of IND, presenting ionizable functional groups (-COOH), can form hydrogen bonds with oily components that exhibit different degrees of polarity (Capryol^®^ 90, Labrafil^®^ M2125 CS, Labrafil^®^M 1944 CS…) [9].

Furthermore, IND exhibited comparable solubility in both SL. No phase separation occurred when mixing Transcutol^®^ P with either SL, therefore, preliminary formulations were prepared to identify the best LL/SL ratio to achieve NLC with superior characteristics. Following a thorough evaluation of the data, both SL provided similar NLC characteristics. These SL are generally recognized as safe (GRAS status) and their use for pharmaceutical products is approved. Therefore, they can be used as lipid carriers for both, solid lipid nanoparticles (SLN) and nanostructured lipid carriers (NLC). Compritol^®^ 888 ATO was selected as the SL for the nanoparticles because it was previously used to develop IND-loaded NLCs [2, 23]. Furthermore, other authors indicated that Precirol^®^ ATO 5 is not a suitable SL for IND encapsulation, as the drug can be easily released [21].

### NLC design and optimization

The characteristics of the formulations obtained in the initial experimental design (N01– N30) are detailed in Supplementary Table 1 (highlighted in grey). This first step aims at selecting the best composition and protocol to obtain IND-loaded NLC within the limits of the experimental design. NLC with sizes ranging from 96.6 nm to 265.1 nm and PDI values between 0.1 and 0.3 were achieved, indicating narrow and homogeneous size distributions [25, 28]. In all cases, the ZP values were negative, ranging between − 3.7 mV and − 16.7 mV. On the other hand, the achieved drug loading felt within the limits of 0.7–3.8%.

**Table 2:** Model A results and quality parameters. Inputs and submodels generated by FormRules^®^ for the different outputs. The most relevant submodels affecting each output are bolded

Output	Submodel	Inputs from FormRules^®^	R^2^	Computed f value	ANOVA freedom degrees	α
Size (nm)	1	Ratio (LL/LS) x [Tween^®^ 80]	85.22	12.81	9 and 20	< 0.01
	2	IND x [Tween^®^ 80]				
PDI	1	IND x [Tween^®^ 80]	75.30	1.60	19 and 10	-
	2	Lecithin x IND				
	3	Ratio (LL/LS)				
	4	Dialysis				
ZP (mV)	1	Ratio (LL/LS) x [Tween^®^ 80]	70.72	7.59	7 and 22	< 0.01
	2	IND				
	3	Lecithin				
	4	Dialysis				
DL (%)	1	IND	87.63	25.97	3 and 11	< 0.01

As indicated in Table 2, FormRules^®^ successfully modelled three out of the four formulation properties with high predictability (R^2^ > 70%) and accuracy (α < 0.01). However, when it comes to PDI, the model struggled to accurately explain variations in this variable. This discrepancy can be attributed to the narrow PDI values distribution suggesting consistently adequate PDI throughout the design space.

**Fig. 3:**
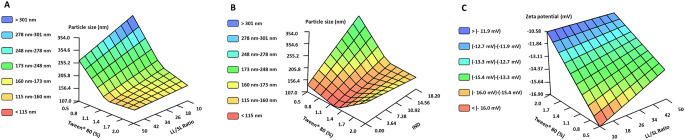
Values predicted by neurofuzzy logic model A for (**A**) the particle size of NLC explained by its submodel 1 and for a constant amount of IND of 16.4 mg; (**B**) the particle size of NLC explained by its submodel 2 and for a constant LL/LS ratio of 50%; and (**C**) zeta potential explained by its submodel 1 and for constant values of lecithin (2%), dialysis time (2 h), and amount of IND (16.4 mg)

According to the obtained models, NLC size is conditioned by three variables: Ratio (LL/SL), Tween^®^ 80 concentration, and amount of IND. Variations in particle size are explained, by the interaction between (LL/SL) ratio and Tween^®^ 80 concentration (Submodel 1 - Fig. 3A) and, on the other hand, by the interaction between the amount of IND and the Tween^®^ 80 concentration (Submodel 2 - Fig. 3B). Being the last submodel the one presenting the strongest effect on NLC size.

FormRules^®^ allows to express the developed models as a set of linguistic rules for each parameter (IF-THEN rules) (Table S2) clarifying the factors conditioning the formulation development [18].

Variations in ZP are mainly explained by the interaction between LL/SL ratio and Tween^®^ 80 concentration (Submodel 1– Fig. 3C). NLC were prepared using negatively charged lipids and a non-ionic surfactant, Tween^®^ 80, which can diminish their surface net charge. As expected, high Tween^®^ 80 concentrations led to less negative ZP values being this difference in surface charge less pronounced at higher LL/SL ratios (Table S2 - Fig. 3C) [32].

Drug loading is directly influenced by the quantity of drug added during the formulation process, higher amounts of IND are required to achieve elevated DL (Table S2). However, this relationship is not linear since the highest IND values led to lower amounts of incorporated drug (Rule 23 Table S2).

Components such as lecithin do not seem to play a decisive role in the characteristics of the NLC. Although lecithin serves as an amphipathic lipid contributing to the stabilization of the NLC dispersion and reducing its aggregation, variations in this parameter, within the studied design space, only result in slight variations in the ZP (Table S2) [32].

INForm^®^ software was successfully employed to model the N01 - N30 database (data not shown) and optimize NLC. The optimization aimed to achieve an IND-loaded NLC formulation with small particle size (< 120 nm), a PDI ranging from 0.2 to 0.3, the lowest surface charge, and the highest DL (> 2%). Table 3 shows the variables selected by INForm’s^®^ genetic algorithms along with the predicted values for the parameters studied to find a compromise solution with maximum desirability.

**Table 3:** Values of selected variables obtained from INForm^®^ optimization, values predicted by the model and those obtained experimentally

Variables		Characteristics	Predicted values	Experimental values
Ratio (LL/SL)	50	Size (nm)	119.6	123.4
Tween^®^ 80 (%)	1.7	ZP (mV)	-15.2	-14.8
Lecithin (mg)	2.0	PdI	0.2	0.2
Dialysis (h)	2.0	DL (%)	3.1	2.5
IND (mg)	16.4			

The obtained NLCs present similar characteristics to those predicted by the INForm^®^ (Table 3). Afterwards, the experimental values of the optimized formulation were added to the database as formulation N31.

Developed NLCs present uniform size distributions (PDI < 0.3) and small particle sizes (< 120 nm), adequate to diffuse trough biological barriers [31]. Moreover, the developed systems presented moderate negative zeta potential (ZP < -10) that can confer stability to the colloidal dispersion [25].

Regarding DL, a high drug content was obtained (> 1%), attributed, to the high solubility of IND in the selected LL together with the NLC unstructured lipid matrix that enhances drug incorporation [4].

Mannose functionalization requires the inclusion of an extra component in the NLC structure, stearylamine (SA). This ingredient enable the reaction of the amino group of SA within the NLC structure with the aldehyde group of mannose, forming a Schiff´s base (-N = CH-) [1].

Therefore, a new process variable was introduced during the formulation procedure, expanding the design space. Consequently, the database was increased with new formulations (N32 to N44 in Table 1), featuring SA amounts within the range of 1.4–7.7 mg.

The database integrating the new formulations characterization results (N01 - N44 in Table S1) was successfully modelled using FormRules^®^ (Model B). The resulting model parameters are presented in Table 4. Similar to previous assessments, the quality of the models was evaluated for predictability (R^2^ > 70% for all formulation properties) and accuracy (α < 0.01 for all formulation parameters).

**Table 4:** Model B results and quality parameters. Inputs and submodels generated by FormRules^®^ for the different outputs. The most relevant submodels for each output are bolded

Output	Submodel	Inputs from FormRules^®^	R^2^	Computed f value	ANOVA freedom degrees	α
Size (nm)	1	[Tween^®^ 80] x SA	91.97	60.77	13 and 69	< 0.01
	2	Ratio (LL/LS)				
PDI	1	[Tween^®^ 80] x SA	77.55	15.89	15 and 69	< 0.01
	2	[Tween^®^ 80] x IND				
ZP (mV)	1	[Tween^®^ 80] x SA	98.00	482.92	8 and 79	< 0.01
	2	IND				
	3	Dialysis				
DL (%)	1	[Tween^®^ 80] x IND	96.78	405.72	6 and 81	< 0.01

**Fig. 4:**
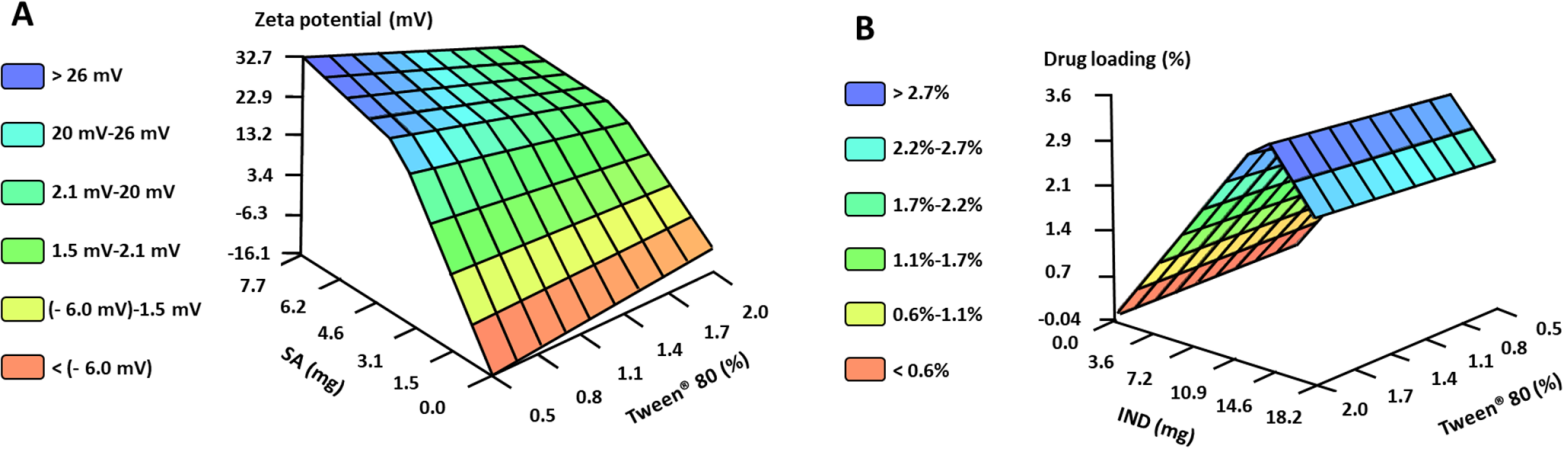
Predicted values by FormRules^®^ for **A**) ZP of NLC explained by its submodel 1 for a constant ratio LL/SL of 50%, and **B**) DL of NLC explained by the interaction between the amount of drug added and the Tween^®^ 80 concentration

As gathered from the inputs selected by FormRules^®^ for model B, the amount of SA has an impact on almost all the parameters studied, highlighting the need to consider this variable for a correct formulation design. The amount of incorporated SA interacts with the Tween^®^ 80 in such a way that the smallest particle sizes are achieved for high values Tween^®^ 80 (> 1.6%) and medium-high values of SA (> 3 mg) (Rule 11 Table S3). SA also influences the PDI but without great relevance since all the values obtained for PDI were lower than 0.3. Furthermore, the amount of incorporated SA extraordinarily conditions the ZP value (Fig. 4A), shifting from negative to positive values after just the addition of a small amount of SA (1 mg). This effect has been previously reported by other authors when developing rifampicin-loaded NLCs [30].

INForm^®^ was used again to model the expanded database (Formulations N01 to N44) (data not shown). This tool was also used to identify the conditions leading to NLC formulations including IND and SA with a small particle size (< 120 nm), a PDI ranging between 0.2 and 0.3, the highest positive surface charge, and a high drug loading (> 2%). Table 5 shows the variables selected by the INForm^®^ model to obtain such formulation, along with the predicted values for the studied parameters.

**Table 5:** Values of the INForm^®^ selected variables (Formulation **IND-NLC-SA**) together with the predicted values and those obtained experimentally before and after storage at 5 °C for 3 months

Variables		Characteristics	Predicted values	Experimental values	Experimental values (after storage)
Ratio (LL/SL)	48.1	Size (nm)	57.6	59.7 ± 0.8	61.7 ± 5.3
Tween^®^ 80 (%)	1.8	ZP (mV)	23.4	22.6 ± 3.6	17.1 ± 2.5
Lecithin (mg)	1.5	PDI	0.3	0.3 ± 0.1	0.3 ± 0.0
Dialysis (h)	3.0	DL (%)	3.4	1.2 ± 0.2	1.2 ± 0.1
IND (mg)	11.0				
SA (mg)	7.7				

Formulations (**IND-NLC-SA**) were experimentally produced under the conditions outlined in Table 5, yielding values similar to those predicted by the model. However, the DL was slightly lower than anticipated, indicating a discrepancy in this parameter that can be attributed to the limited number of loaded formulations with SA in the database.

Furthermore, the properties of **IND-NLC-SA** after a 3-month storage period were re-analyzed to evaluate the stability of the NLC. The formulated **IND-NLC-SA** were stable over the specified duration, with no statistically significant differences observed in the NLC characteristics, including size, PDI, ZP and DL over time.

### NLC functionalization with mannose

Nuclear magnetic resonance (NMR) spectroscopy analysis enables the detailed structural chemical characterization of different materials including carbohydrates [12].

**Fig. 5:**
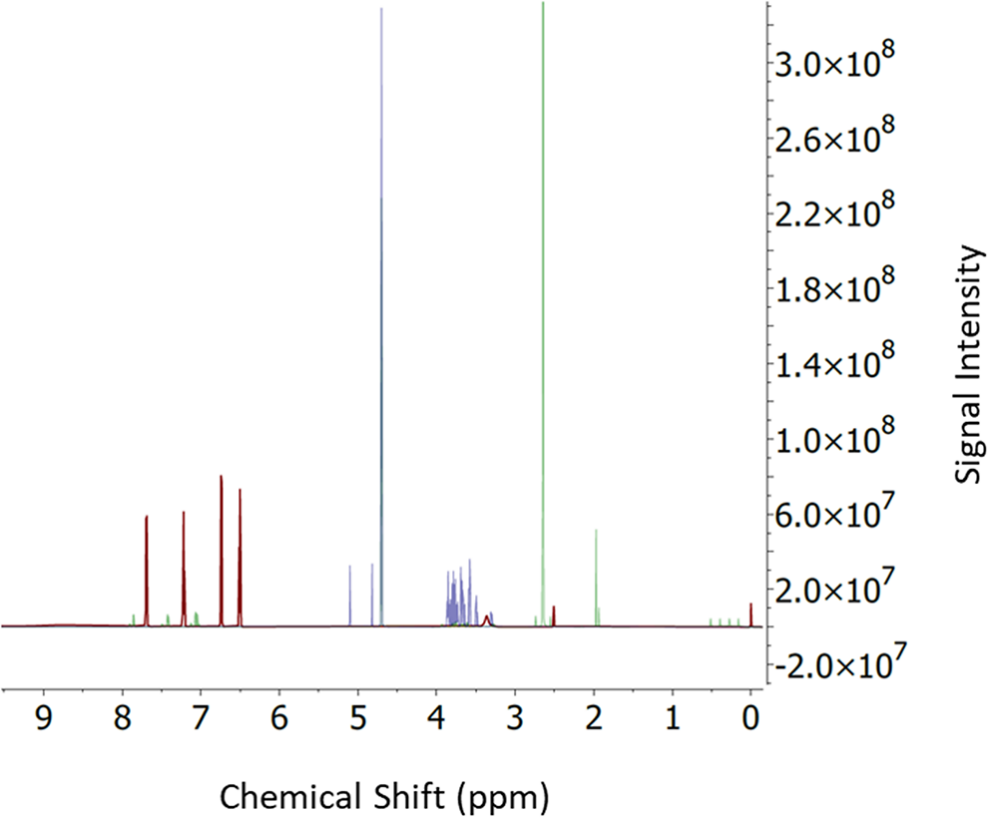
^1^H NMR spectrum of D-(+)-Mannose (violet spectrum) overlapped with the product derived from the Maillard reaction to fluorescently label mannose (green spectrum), and with the anthranilic acid (red spectrum)

Figure 5 shows the ^1^H NMR spectrum of the D-(+)-mannose (violet) overlapped with the fluorescently labelled mannose derived from the Maillard reaction (green) and with free anthranilic acid (red). These findings suggest that mannose was bounded to the fluorophore observing a shift in the peaks of anthranilic acid after conjugation. Moreover, two additional peaks could be observed for the labeled mannose coming from the acetate buffer used for dilution and purification previous to lyophilization.

The use of fluorescently labelled mannose allowed the NLC functionalization efficiency calculation, which is expressed as the percentage of the added component incorporated to the NLC surface. Moreover, this approach is useful to optimize the functionalization procedure. As far as we know, this method has not been used previously. Based on literature, different SA: MAN proportions and incubation times have been selected to cover most of the previously reported settings. Table 6 exhibits the **IND-NLC-SA** mannose functionalization efficiency in the different conditions studied, along with the naïve NLC characteristics (particle size, PDI, ZP).

**Table 6:** Results of mannose-functionalization efficiency of **IND-NLC-SA** optimal formulation and NLC characteristics after their incubation with D-(+)-mannose (50 mM) at various SA: MAN molar ratio and incubation times

SA: MAN molar ratio	Incubation time (h)	Functionalization efficiency (%)	Size (nm)	PDI	ZP (mV)
1:2	24	86.59 ± 4.21	73.94 ± 2.24	0.27 ± 0.03	21.69 ± 3.59
1:2	48	85.15 ± 7.26	70.88 ± 0.58	0.26 ± 0.03	23.34 ± 3.06
1:2	72	86.24 ± 6.23	71.98 ± 1.24	0.26 ± 0.03	24.84 ± 4.62
1:3	24	71.98 ± 16.45	74.22 ± 2.26	0.27 ± 0.01	23.12 ± 2.45
1:3	48	70.71 ± 20.01	73.32 ± 0.87	0.27 ± 0.01	23.41 ± 0.08
1:3	72	67.85 ± 21.15	71.54 ± 2.87	0.27 ± 0.01	25.24 ± 4.15
1:5	24	65.83 ± 13.40	71.80 ± 3.08	0.25 ± 0.02	23.50 ± 1.75
1:5	48	61.62 ± 17.85	72.91 ± 1.10	0.26 ± 0.02	24.91 ± 0.26
1:5	72	62.48 ± 16.38	72.91 ± 2.88	0.26 ± 0.01	24.06 ± 0.53
Non-functionalized NLCs **(IND-NLC-SA)**			59.72 ± 0.84	0.30 ± 0.05	22.62 ± 3.56

As depicted in Table 6, the functionalization process following all the studied protocols results in NLC with significantly higher particle size (*p < 0.0001*) aligning with findings reported by previous researchers [17]. Despite no statistically significant changes were observed in the ZP of naïve and functionalized NLC, as a general trend higher ZP values were obtained in functionalized NLC.

**Fig. 6:**
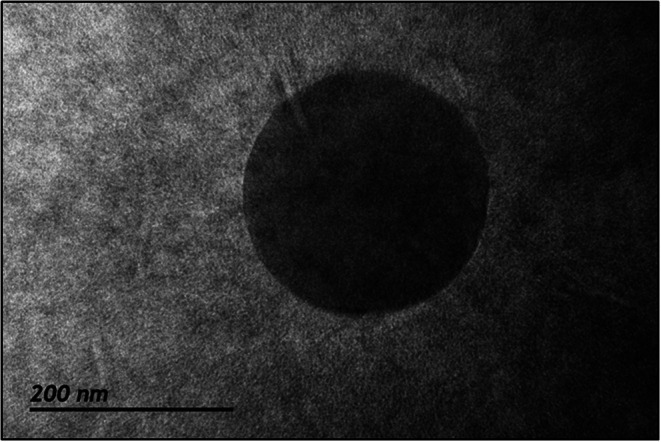
Transmission electron micrograph of **IND-NLC-SA-MAN** formulation functionalized with D-(+)-mannose at 1:2 SA: MAN molar ratio for 72 h

No statistically significant differences were found in the NLC properties between variable incubation times at any of the ratios used. Moreover, elevated SA: MAN molar ratios (molar ratio > 1:3) contributed to amplified variability in NLC functionalization efficiency. On the other hand, the functionalization efficiency exceeds 85% for 1:2 ratios, suggesting that the optimal formulation (**IND- NLC-SA**), developed through the sequential procedure (Table 5), is well suited for mannose functionalization at this ratio. This formulation ensures a uniform particle size distribution (PDI 0.3), small particle sizes (≈ 70 nm), and a high positive zeta potential (> 20 mV) that should ensure stability [25]. The morphology of the mannose functionalized nanoparticles at this ratio (1:2) using a 72-hour incubation (**IND-NLC-SA*-*MAN**) is shown in Fig. 6. The obtained nanoparticles depicted a spherical shape with a smooth surface.

These mannosylated NLCs must exhibit enhanced characteristics for macrophages uptake, a phenomenon demonstrated by previous researchers with mannosylated dendrimers or SLNs. The presence of mannose on the surfaces of those DDS enhanced macrophage recognition and internalization, underlining the importance of optimizing mannosylation for improved macrophages interactions [13, 17, 22].

## Conclusions

This work highlights the remarkable capacity of hybrid AI tools to facilitate formulation design through sequential development processes. The results demonstrate it is feasible to model databases containing a significant number of variables and incorporate additional ones during the process. The integration of AI technologies enables a more accessible processes understanding and production conditions optimization with minimal experimentation, thus reducing time and cost. The use of these models has facilitated the design of stable NLCs through a robust production method, ensuring the particles have suitable characteristics for being recognized and internalized by macrophages. Its composition allows efficient functionalization with mannose, offering a promising strategy for treating diseases associated with inflammation. Furthermore, to our knowledge, this study presents the first instance in which a fluorescently labelled carbohydrate was employed to assess the functionalization efficiency of mannosylated drug delivery systems.

## Electronic supplementary material

Below is the link to the electronic supplementary material.

Supplementary Material 1
