# Supplementary material for: Engineering mannose-functionalized nanostructured lipid carriers by sequential design using hybrid artificial intelligence tools
Source: Drug Deliv Transl Res. 2024 May 9;15(1):343–54. doi: 10.1007/s13346-024-01603-z (PMC11615115; doi:10.1007/s13346-024-01603-z)
Supplement: Supplementary file 1 — Supplementary Material 1 [file 13346_2024_1603_MOESM1_ESM.docx]

**SUPPLEMENTARY MATERIAL**

Table S1. Characteristics of the NLC studied. Grey area corresponds to the formulations used for the Model A. The complete database was used for Model B.

| **Formulations** | **Formulation -characteristics** | | | |
| --- | --- | --- | --- | --- |
|  | **Particle size (nm)** | **ZP (mV)** | **PDI** | **DL (%)** |
| N01 | 124.2 | -15.2 | 0.3 | - |
| N02 | 160.3 | -9.1 | 0.1 | - |
| N03 | 148.3 | -10.5 | 0.2 | - |
| N04 | 139.8 | -10.5 | 0.2 | - |
| N05 | 127.8 | -14.5 | 0.2 | - |
| N06 | 174.1 | -7.6 | 0.1 | - |
| N07 | 185.8 | -15.4 | 0.3 | - |
| N08 | 171.5 | -7.2 | 0.2 | - |
| N09 | 98.3 | -11.6 | 0.2 | - |
| N10 | 168.4 | -7.2 | 0.1 | - |
| N11 | 111.9 | -3.7 | 0.2 | - |
| N12 | 232.1 | -14.6 | 0.2 | - |
| N13 | 182.2 | -6.4 | 0.2 | - |
| N14 | 233.8 | -15.6 | 0.3 | - |
| N15 | 128.8 | -11.4 | 0.2 | - |
| N16 | 179.7 | -16.1 | 0.2 | - |
| N17 | 161.3 | -11.0 | 0.2 | - |
| N18 | 149.2 | -12.8 | 0.2 | - |
| N19 | 172.5 | -12.4 | 0.2 | - |
| N20 | 184.0 | -15.3 | 0.2 | - |
| N21 | 162.6 | -9.9 | 0.2 | - |
| N22 | 230.6 | -15.2 | 0.2 | - |
| N23 | 165.8 | -8.5 | 0.2 | - |
| N24 | 104.9 | -12.3 | 0.2 | - |
| N25 | 170.6 | -10.2 | 0.2 | - |
| N26 | 122.4 | -9.4 | 0.2 | - |
| N27 | 240.1 | -16.7 | 0.2 | - |
| N28 | 181.1 | -11.3 | 0.2 | - |
| N29 | 265.1 | -16.4 | 0.2 | - |
| N30 | 151.1 | -12.9 | 0.2 | - |
| N01 | 122.5 | -7.9 | 0.3 | - |
| N02 | 160.5 | -8.0 | 0.1 | - |
| N03 | 161.0 | -13.0 | 0.2 | - |
| N04 | 149.9 | -10.3 | 0.2 | - |
| N05 | 124.1 | -13.4 | 0.2 | - |
| N06 | 211.3 | -10.8 | 0.1 |  |
| N07 | 203.1 | -12.9 | 0.3 | - |
| N08 | 194.1 | -10.8 | 0.2 | - |
| N09 | 96.6 | -10.9 | 0.2 | - |
| N10 | 190.2 | -11.1 | 0.1 | - |
| N11 | 117.7 | -11.6 | 0.2 | - |
| N12 | 224.3 | -13.8 | 0.2 | - |
| N13 | 219.5 | -10.7 | 0.2 | - |
| N14 | 220.9 | -14.7 | 0.3 | - |
| N15 | 142.2 | -12.2 | 0.2 | - |
| N16 | 179.7 | -14.2 | 0.2 | 1.0 |
| N17 | 169.7 | -10.9 | 0.2 | 3.8 |
| N18 | 153.0 | -13.4 | 0.2 | 0.9 |
| N19 | 187.3 | -12.9 | 0.2 | 1.6 |
| N20 | 184.0 | -13.9 | 0.2 | 1.4 |
| N21 | 185.0 | -11.6 | 0.2 | 3.4 |
| N22 | 230.6 | -15.2 | 0.2 | 0.7 |
| N23 | 187.5 | -11.0 | 0.2 | 1.3 |
| N24 | 148.6 | -12.6 | 0.2 | 3.4 |
| N25 | 196.8 | -11.4 | 0.2 | 2.9 |
| N26 | 122.4 | -12.2 | 0.2 | 2.0 |
| N27 | 248.0 | -16.0 | 0.2 | 1.3 |
| N28 | 117.8 | -14.6 | 0.2 | 2.7 |
| N29 | 257.1 | -15.3 | 0.2 | 1.4 |
| N30 | 151.1 | -13.1 | 0.2 | 2.7 |
| N31 | 121.6 | -14.0 | 0.2 | - |
| N32 | 322.7 | 28.5 | 0.2 | - |
| N33 | 261.9 | 36.9 | 0.2 | - |
| N34 | 234.9 | 30.3 | 0.3 | - |
| N35 | 229.1 | 33.3 | 0.3 | - |
| N36 | - | 2.1 | - | - |
| N37 | 334.2 | 10.1 | 0.3 | - |
| N38 | 159.4 | 15.6 | 0.2 | - |
| N39 | 130.4 | -11.2 | 0.2 | - |
| N40 | 86.8 | 12.2 | 0.3 | - |
| N41 | - | -0.7 | - | - |
| N42 | - | 2.2 | - | - |
| N43 | 93.7 | 13.8 | 0.4 | - |
| N44 | 78.4 | 16.7 | 0.4 | - |
| N31 | 123.4 | -14.8 | 0.2 | 2.5 |
| N32 | 320.0 | 26.0 | 0.3 | 1.0 |
| N33 | 303.6 | 34.6 | 0.2 | 0.7 |
| N34 | 206.0 | 25.0 | 0.2 | 1.7 |
| N35 | 200.6 | 31.2 | 0.2 | 1.3 |
| N36 | - | -1.0 | - | 3.4 |
| N37 | 347.2 | 7.3 | 0.3 | 2.7 |
| N38 | 174.6 | 12.5 | 0.2 | 3.2 |
| N39 | 153.6 | -10.8 | 0.2 | 3.6 |
| N40 | 92.0 | 10.5 | 0.3 | 3.4 |
| N41 | 609.3 | -5.6 | 0.3 | 2.6 |
| N42 | - | -3.6 | - | 3.1 |
| N43 | 75.1 | 11.6 | 0.3 | 3.0 |
| N44 | 65.1 | 12.0 | 0.3 | 3.3 |

Table S2. Rules generated by FormRules® (Model A) for the parameters size, surface charge (ZP), and drug loading (DL) (membership degrees in parentheses). In blue and red, the combinations of variables that lead the highest and lowest value of the parameter studied.

| **Rule** | **Property: Size (nm)** |  |  |
| --- | --- | --- | --- |
|  | **SubModel:1** |  |  |
| 1 | IF Ratio (LL/SL) is LOW AND Tween® 80 (%) is LOW | THEN Size (nm) is | HIGH (1.00) |
| 2 | IF Ratio (LL/SL) is LOW AND Tween® 80 (%) is HIGH | THEN Size (nm) is | HIGH (0.56) |
| 3 | IF Ratio (LL/SL) is HIGH AND Tween® 80 (%) is LOW | THEN Size (nm) is | LOW (0.61) |
| 4 | IF Ratio (LL/SL) is HIGH AND Tween® 80 (%) is HIGH | THEN Size (nm) is | LOW (0.55) |
|  | **SubModel:2** |  |  |
| 5 | IF Tween® 80 (%) is LOW AND IND is LOW | THEN Size (nm) is | LOW (0.93) |
| 6 | IF Tween® 80 (%) is LOW AND IND is HIGH | THEN Size (nm) is | HIGH (1.00) |
| 7 | IF Tween® 80 (%) is MID AND IND is LOW | THEN Size (nm) is | LOW (1.00) |
| 8 | IF Tween® 80 (%) is MID AND IND is HIGH | THEN Size (nm) is | LOW (0.70) |
| 9 | IF Tween® 80 (%) is HIGH AND IND is LOW | THEN Size (nm) is | HIGH (0.74) |
| 10 | IF Tween® 80 (%) is HIGH AND IND is HIGH | THEN Size (nm) is | LOW (0.72) |
|  |  |  |  |
|  | **Property: ZP (mV)** |  |  |
|  | **SubModel:1** |  |  |
| 11 | IF Tween® 80 (%) is LOW AND Ratio (LL/SL) is LOW | THEN ZP (mV) is | LOW (1.00) |
| 12 | IF Tween® 80 (%) is LOW AND Ratio (LL/SL) is HIGH | THEN ZP (mV) is | LOW (1.00) |
| 13 | IF Tween® 80 (%) is HIGH AND Ratio (LL/SL) is LOW | THEN ZP (mV) is | HIGH (1.00) |
| 14 | IF Tween® 80 (%) is HIGH AND Ratio (LL/SL) is HIGH | THEN ZP (mV) is | HIGH (1.00) |
|  | **SubModel:2** |  |  |
| 15 | IF IND (mg) is LOW | THEN ZP (mV) is | HIGH (1.00) |
| 16 | IF IND (mg) is HIGH | THEN ZP (mV) is | LOW (1.00) |
|  | **SubModel:3** |  |  |
| 17 | IF Lecithin (mg) is LOW | THEN ZP (mV) is | LOW (0.86) |
| 18 | IF Lecithin (mg) is HIGH | THEN ZP (mV) is | HIGH (0.67) |
|  | **SubModel:4** |  |  |
| 19 | IF Dialysis (h) is LOW | THEN ZP (mV) is | HIGH (0.70) |
| 20 | IF Dialysis (h) is HIGH | THEN ZP (mV) is | LOW (0.89) |
|  |  |  |  |
|  | **Property: DL (%)** |  |  |
|  | **SubModel:1** |  |  |
| 21 | IF IND (mg) is LOW | THEN DL (%) is | LOW (0.89) |
| 22 | IF IND (mg) is MID | THEN DL (%) is | HIGH (0.84) |
| 23 | IF IND (mg) is HIGH | THEN DL (%) is | HIGH (0.74) |

Table S3. Rules generated by FormRules® (Model B) for the parameters size, surface charge (ZP), and drug loading (DL) (membership degrees in parentheses). In blue and red, the combinations of variables that lead the highest and lowest value of the parameter studied.

| **Rule** | **Property: Size (nm)** | |  |  |
| --- | --- | --- | --- | --- |
|  | **SubModel:1** | |  |  |
| 1 | IF Tween % is LOW AND SA (mg) is LOW_1 | | THEN Size (nm) is | LOW (1.00) |
| 2 | IF Tween % is LOW AND SA (mg) is MID_2 | | THEN Size (nm) is | LOW (1.00) |
| 3 | IF Tween % is LOW AND SA (mg) is MID_3 | | THEN Size (nm) is | LOW (1.00) |
| 4 | IF Tween % is LOW AND SA (mg) is HIGH_4 | | THEN Size (nm) is | LOW (1.00) |
| 5 | IF Tween % is MID AND SA (mg) is LOW_1 | | THEN Size (nm) is | LOW (1.00) |
| 6 | IF Tween % is MID AND SA (mg) is MID_2 | | THEN Size (nm) is | HIGH (1.00) |
| 7 | IF Tween % is MID AND SA (mg) is MID_3 | | THEN Size (nm) is | HIGH (1.00) |
| 8 | IF Tween % is MID AND SA (mg) is HIGH_4 | | THEN Size (nm) is | LOW (1.00) |
| 9 | IF Tween % is HIGH AND SA (mg) is LOW_1 | | THEN Size (nm) is | LOW (1.00) |
| 10 | IF Tween % is HIGH AND SA (mg) is MID_2 | | THEN Size (nm) is | HIGH (1.00) |
| 11 | IF Tween % is HIGH AND SA (mg) is MID_3 | | THEN Size (nm) is | LOW (1.00) |
| 12 | IF Tween % is HIGH AND SA (mg) is HIGH_4 | | THEN Size (nm) is | LOW (1.00) |
|  | **SubModel:2** | |  |  |
| 13 | IF Ratio (LL/SL) is LOW | | THEN Size (nm) is | HIGH (1.00) |
| 14 | IF Ratio (LL/SL) is HIGH | | THEN Size (nm) is | HIGH (1.00) |
|  |  | |  |  |
|  | **Property: ZP (mV)** | |  |  |
|  | **SubModel:1** | |  |  |
| 15 | IF SA (mg) is LOW AND Tween® 80 (%) is LOW | | THEN ZP (mV) is | LOW (1.00) |
| 16 | IF SA (mg) is LOW AND Tween® 80 (%) is HIGH | | THEN ZP (mV) is | LOW (1.00) |
| 17 | IF SA (mg) is MID AND Tween® 80 (%) is LOW | | THEN ZP (mV) is | HIGH (1.00) |
| 18 | IF SA (mg) is MID AND Tween® 80 (%) is HIGH | | THEN ZP (mV) is | LOW (0.73) |
| 19 | IF SA (mg) is HIGH AND Tween® 80 (%) is LOW | | THEN ZP (mV) is | HIGH (1.00) |
| 20 | IF SA (mg) is HIGH AND Tween® 80 (%) is HIGH | | THEN ZP (mV) is | LOW (0.73) |
|  | **SubModel:2** | |  |  |
| 21 | IF IND (mg) is LOW | | THEN ZP (mV) is | HIGH (0.69) |
| 22 | IF IND (mg) is HIGH | | THEN ZP (mV) is | HIGH (0.50) |
|  | **SubModel:3** | |  |  |
| 23 | IF Dialysis (h) is LOW | | THEN ZP (mV) is | HIGH (0.66) |
| 24 | IF Dialysis (h) is HIGH | | THEN ZP (mV) is | HIGH (0.53) |
|  |  | |  |  |
|  | **Property: DL (%)** |  | |  |
|  | **SubModel:1** |  | |  |
| 25 | IF IND (mg) is LOW AND Tween® 80 (%) is LOW | THEN DL (%) is | | LOW (1.00) |
| 26 | IF IND (mg) is LOW AND Tween® 80 (%) is HIGH | THEN DL (%) is | | LOW (0.99) |
| 27 | IF IND (mg) is MID AND Tween® 80 (%) is LOW | THEN DL (%) is | | HIGH (0.87) |
| 28 | IF IND (mg) is MID AND Tween® 80 (%) is HIGH | THEN DL (%) is | | HIGH (0.98) |
| 29 | IF IND (mg) is HIGH AND Tween® 80 (%) is LOW | THEN DL (%) is | | HIGH (0.62) |
| 30 | IF IND (mg) is HIGH AND Tween® 80 (%) is HIGH | THEN DL (%) is | | HIGH (0.69) |
